# Coronary artery bypass grafting in young patients - insights into a distinct entity

**DOI:** 10.1186/s13019-015-0266-1

**Published:** 2015-05-01

**Authors:** Felix Fleissner, Gregor Warnecke, Serghei Cebotari, Saad Rustum, Axel Haverich, Issam Ismail

**Affiliations:** Division of Cardiac, Thoracic, Transplantation and Vascular Surgery, Hannover Medical School, Hannover, Germany

**Keywords:** CABG, Arterial grafts, Extracorporeal life support (ECLS), Myocardial ischemia, Ischemic cardiomyopathy

## Abstract

**Objectives:**

Coronary artery bypass grafting (CABG) is the ‘Gold Standard’ for patients with multiple vessel coronary artery disease (CAD). Younger patients presenting with coronary artery disease requiring surgery may represent a distinct subgroup with the main goal for coronary revascularization being long term patency of the performed grafts to improve outcome.

**Methods:**

Between January 2010 and August 2013, 126 patients below the age of 50 years underwent CABG for CAD in our hospital. We retrospectively analyzed the perioperative data and evaluated patients’ outcome.

**Results:**

In 25% of the patients CABG was performed as an emergency procedure for STEMI or NSTEMI within 36 hours. Another 27% of the patients were operated urgently for unstable angina or myocardial infarction within the last weeks and only 48% of the patients were purely elective cases. We performed only venous bypass grafts in 12%, total arterial revascularisation in 52% of all cases and combined venous and arterial revascularization in 43%. Six patients needed cardiac support using an extracorporeal membrane oxygenation (Mortality n = 1 out of 6) and 17 patients received an intraaortic ballon pump perioperativly. Patients received 2.8 ± 1 bypass grafts overall. Overall in-hospital mortality in this cohort was low with 1% (n = 1).

**Conclusions:**

In conclusion, the majority of the young patients below the age of 50 years present urgently for operative revascularization. Besides the potential advances regarding long term patency using total arterial revascularization, only about half of the young patients are feasible for this approach. Overall early outcome in this group is excellent with mortality below one percent.

## Background

Coronary artery bypass grafting (CABG) remains the Gold standard for excessive coronary artery disease involving three vessels or the left main stem [1]. CABG is more durable than percutaneous coronary intervention, especially when using arterial grafts only [2-4]. There is abundant literature regarding risks of elderly patients undergoing cardiac surgery [5-7]. However, reports about younger patients undergoing CABG are sparse. D’Errigo et al. recently reported the multicenter data concerning patients below 50 years of age receiving CABG with a mortality rate of 0.9% overall [8]. However, no details about the used grafts were given in that study. A low postoperative mortality rate has also been reported by Khawaja et al. [9] in patients aged <50 years treated by percutaneous coronary intervention (0.86%). However, percutaneous coronary interventions were performed in 41% of cases in patients with single vessel coronary artery disease, which is significantly different from surgical series [8].

Patients who would benefit most from prolonged patency of total artery revascularization are the young patients. In this study we therefore sought to further evaluate patients under the age of 50 years receiving coronary artery bypass grafting.

## Methods

We retrospectively analysed all consecutive patients below the age of 50 years receiving CABG at our clinic from January 2010 until August 2013. The Ethics Committee of Hanover Medical School approved this study. Informed consent was obtained from the patient for the publication of this report.

### Operative technique

A full median sternotomy was performed (except one patient receiving an upper hemisternotomy due to a high grade stenosis of the right coronary artery of anomalous origin). We used cardiopulmonary support in most cases (n = 125), except for the patient receiving the upper hemisternotomy.

### Total arterial revascularization (TAR)

*A*fter a full median sternotomy the LITA was harvested in no touch technique with minimal trauma as pedicled graft and treated with papaverine solution prior to use. RA was harvested from the non-dominant arm. We performed duplex sonography to assure open palmar arches. The T-graft was performed prior to initiating CPB between the RA and the LITA using an 8–0 prolene suture. CBP was initiated after completion of the T graft.

### Combined venous and arterial revascularization

We usually used the LITA and the saphenous vein as grafts. The LITA was most frequently anastomosed to the LAD or D1, whereas the saphenous vein was used for up to three sequential anastomoses. The venous only revascularization was achieved using the saphenous vein as aortocoronary bypass anastomosed to the acending aorta as standardized.

CPB was instituted with cannulation of the ascending aorta and a single two-stage right atrial cannulation. Standard bypass management included membrane oxygenators, arterial line filters, and non-pulsatile flow (or pulsatile flow for patients kidney failure) with a mean arterial pressure greater than 50 mm Hg. The myocardium was protected by using intermittent antegrade cold blood cardioplegia or crystalloid solution. Anticoagulation was achieved using 300 U/kg of heparin. If required, heparin was supplemented to maintain the activated clotting time above 450 s and was fully reversed with protamine at the end of the procedure. Patients received intravenous nitroglycerin infusions for the first 24 h if feasible. Inotropic agents were chosen by the haemodynamic state. Other routine medications included daily aspirin (300mg Aspirin i.v. 6 hour postoperatively unless bleeding occurred, 100mg Aspirin p.o. the first postoperative day) and resumption of cholesterol-lowering agents and β-blockers unless contraindicated beginning during the early postoperative course.

#### Statistical analysis

Statistical analysis was performed using SPSS 22 package (SPSS Inc, Chicago, Il, USA). The data were shown as mean ± SD. Comparison of groups was performed using unpaired t-test or ANOVA with statistical significance assumed for p values <0.05.

## Results

### Patients

A total of 126 CABG cases in patients below the age of 50 years were performed. Twenty five percent (n = 30) of the operations were emergencies, Twenty seven percent (n = 35) of the patients were operated urgently and only 48% (n = 61) of the patients were elective cases. 52 patients recently had a myocardial infarction (<30 days) of which 18 patients presented with myocardial infarction under 12 hours prior to the operation.

Median age was 46.98 (range from: 24–49), 81% (n = 102) of the patients were male. Patients usually presented with multiple risk factors. Arterial hypertension was present in 77%, nicotine abuse in 79% and hyperlipidaemia in 71% of patients. Obesity was present in 34% of cases, diabetes mellitus in 25% of cases and a familial pre-disposition was reported in only 20% of cases. The overall pre-operative left ventricular ejection fraction (LV-EF) was 55.5% (±13.8%). However, 12% of patients presented with a poor LV-EF below 35%. Only two patients were cardiac re-dos (one AVR and one CABG). Nine patients recently underwent CPR in the pre-operative course due to ventricular fibrillation. 36 Patients had already undergone PTCA/Stent implantation before they were admitted to cardiac surgery.

Of these 36 patients who received an intervention prior to CABG, 3 patients presented with iatrogenic dissections in the target vessels and were subsequently referred to CABG, 4 patients had undergone an unsuccessful PCI attempt in one or two target vessels prior to referral to operative revascularization. 10 patients presented with in-stent re-(re) stenosis in stented vessels. Only one patient received a PCI (without stent implantation) of the culprit lesion (RPLA) directly prior to surgery as a bridging therapy to surgical revascularization in acute myocardial infarction (STEMI). One patient was reanimated extrahospitally and received a lysis therapy. The patient received a coronary angiogram after admission to the hospital which showed a 3 vessel disease. Another patient with an iatrogenic dissection of the LAD received ReoPro directly prior to operation as a rescue attempt.

66% (n = 83) of patients were symptomatic prior to the operation (NSTEMI, STEMI and unstable angina).

Six patients presented with severe mitral valve regurgitation du to ischemic papillary muscle rupture. 12 Patients had renal insufficiency preoperatively, of those 3 patients were on dialysis. For further pre-operative details, please refer to Table 1.

**Table 1:** **Patient’s characteristics**

Patient’s characteristics:	
Age, years	46.98 (range from: 24–49)
Sex, male (%)	102 (81)
Left ventricular ejection fraction in % (± SD)	55.5 (±13.8)
Poor LV-EF (35% or below)	15 (12)
Creatinine (± SD)	85.6 (±66.9)
CK U/l (± SD)	375.9 (±1072)
CK-MB U/l (± SD)	45.4 (±96.1)
Pre-op renal insufficiancy (%)	12 (10)
Pre-op dialysis (%)	3 (2)
Arterial hypertension (%)	97 (77)
Nicotine abuse (%)	99 (79)
Hyperlipidaemia (%)	89 (71)
Diabetes mellitus (%)	25 (20)
Obesity (%)	43 (34)
Emergency (%)	30 (25)
Urgent (%)	35 (27)
Elective (%)	61 (48)
Non-ST-elevation acute coronary syndrome (%)	25 (20)
Recent STEMI (%)	25 (20)
Unstable angina (%)	33 (26)
Stable angina (%)	43 (34)
Previous PTCA/Stent (%)	36 (28)
Unsuccessful previous PTCA/Stent attempt (%)	3 (2)
IABP	17 (14)
ECLS	6 (5)
Cardiac Re-do	2 (2)

Continuous variables are presented with the standard deviation or median with range (age), categoric variables are presented as number (%).

LV-EF (Left-ventricular ejection fraction), CK (creatinine kinase), CK-MB (muscle-brain creatinine kinase), STEMI (ST-elevation myocardial infarction), IABP(intraaortic ballon pump), ECLS (Extracorporal Life Support System), PTCA (percutaneous transluminal coronary angioplasty).

Operation, cardiopulmonary bypass and clamp times were 203.96 ± 58.51 min 88.70 ± 39.97 min and 49.95 ± 22.63 min, respectively. ICU stay and overall hospital stay were 2.23 ± 3.54 days and 8.64 ± 4.57 days respectively (See Table 2). In-hospital and 30 day mortality was 0.9% (n = 1). Six patients needed Extracorporal Life Support System (ECLS) (See Table 3) and 17 patients received peri-operative intraaortic ballon pump (IABP) support. One patient died during postoperative course. This patient presented in cardiogenic shock due to STEMI, was switched from IABP support to ECLS support and died during post-operative course despite all efforts in multi-organ failure. Retrospectively, this patient might have benefited from initial ECLS support instead of IABP support. The other five patients could be weaned successfully from ECLS support. Concomitant procedures included mitral valve repair or replacement in six patients. One patient received an aortic valve replacement due to severe aortic insufficiency and one patient received a carotid endarterectomy. Three patients received a DOR plasty due to left ventricular aneurysms.

**Table 2:** **Operative data**

Operative data:	
Operation time min (± SD)	203.96 (±58.51)
Cardiopulmonary bypass times min (± SD)	88.71 (±39.97)
Clamp times min (± SD)	45.95 (±22.63)
Of-pump procedures	1 (1)
Venous bypass grafts (exclusivly)	15 (12)
Total arterial revascularization	66 (52)
Arterial and venous revascularization	43 (34)
Number of performed bypass grafts n (± SD)	2.87 (±0.92)
*Concomittant procedures*	
Mitral valve repair	3
Mitral valve replacement	3
Aortic valve replacement	1
DOR Plasty	3
Carotid TEA	1

Continuous variables are presented with the standard deviation; categoric variables are presented as number (%). Carotid TEA (thromboendarterectomy).

**Table 3:** **ECLS subgroup analysis**

Patient	Presentation	Indication for ECLS	ECLS support	Surgery	Postoperative course	Outcome
1	NSTEMI, CA: Bifurcation stenosis of LAD/D1	difficult weaning from CPB	d0-d2	LIMA-LAD,-D1	ECMO explanation D3, uneventful postoperative course	LV-EF at discharge 50%
2	Present to referring hospital with angina, CA: RCA 100%, CX: procimal 90%, LAD; 100%, unsuccessful attempt for PTCA/Stentimplantation of the CX with iatrogenic dissection, rescue attempt with ReoPro, CPR during postoperative course, operation the same day as admission to primary hospital	difficult weaning from CPB under running IABP, ECLS intraoperativly	d0-d9	ACVB-LAD, ACVB-PLA1-PLA3, RIVP	dialysis during postoperative course, recovered POD 17, prolonged ventilation with tracheotomy, removed POD 23, Re-Thoracotomy POD 1, POD 12 due to haematothorax with pericardial tamponade	LV-EF POD 1: 20% under ECLS support), LV-EF prior to discharge: 50%
3	STEMI, CA: LAD 100% proximal, CX, PLA 1 90%, RCA 100%, Patient in cardiogenic shock with unstable hameodynamic under catecholamines, immediate referral to OR	cardiogenic shock, difficult weaning from CPB under IABP support	d0-d12	LIMA-LAD, ACVB-RIVP-PLA	dialysis during postoperative course, recovery day 14,	LV-EF POD 1 under ECLS 25%, LV-EF prior to discharge 35%
4	Unstable angina, CA: LAD 90%, Cx 100%, RCA 100%, cardiac decompensation with peripheral oedema and septic ulcers, multiple sclerosis, severe obesity	cardiogenic shock, difficult weaning from CPB under IABP support	d0-d7	LIMA-LAD-D1, radial artery PLA-RIVP (septic ulcers, no vein grafts available)	Dialysis during postoperative course, compartment syndrome left lower leg, re-thoracotomy due to tamponade, septic shock	death POD 7
5	presentation with beginning cardiac decompensation and respiratory insufficiency, CA: LAD 100%, Cx 90, RCA 90%, ECLS support in reffering hospital prior to transport, Echo: LV-EF 45%, MI III (rupture of posteromedian papillary muscle)	cardiogenic shock	d-1-d4	MVR (SJM, 29 mm), ACVB-LAD, ACVB-PLA1-RIVP	Dialysis during postoperative course, recovered POD 5, short CPR on POD 5, ICD implantation POD 30	LV-EF at discharge 65% mechanical prosthesis in mitral position with no PVL and normal function
6	Angina and Dyspnoea in referring hospital, CA: LAD 40%, PLA 2 90%, RIVP 70%, MI III	difficult weaning from CPB, ECLS intraoperativly	d0-d8	MVR (anuloplasty with ring), ACVB-PLA-RIVP	Dialysis once (POD 1), prolonged ventilation with tracheotomy until POD 27	LV-EF prior to discharge 40%

Clinical presentation, performed surgery, postoperative course and outcome of the six patients requiring ECLS. ECLS (Extracorporal Life Support System) NSTEMI (Non-ST-Elevated Myocardial Infarction), STEMI (ST-elevation myocardial infarction), MI (mitral valve insufficiency), IABP (intraaortic ballon pump), CPB (Cardiopulmonary bypass) POD (Postoperative day), LV-EF (Lef-ventricular ejection fraction), PVL (paravalvular leakage).

The left internal thoracic artery (LITA) was used in 111 cases (88%) and either anastomosed to the LAD, LAD and D1, or to the RIM. The right internal thoracic artery (RITA) was used in 8 cases (6%). The radial artery (RA) as a T-graft was used in 51 cases and as a free graft in two cases. In total, the RA was used in 53 cases (42%).

In case of a total arterial revascularisation, combined venous and arterial revascularisation and venous revascularisation only, 2.6 (±0.89), 3.19 (±0.93) and 2.75 (±1.39) bypass grafts were anastomosed, respectively.

We performed venous bypass grafts in 12% (n = 15), total arterial revascularisation (using mainly the left internal thoracic artery with the radial artery as T-graft) in 52% (n = 62) of all cases and combined venous and arterial revascularization in 43% (n = 34).

**Figure 1:**
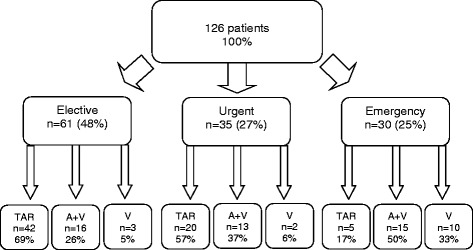
Flow chart of the 126 pts. receiving coronary artery bypass grafting. TAR: total artery revascularisation, A + V: combined venous and arterial revascularisation, V: venous grafts only.

Total arterial revascularisation was performed in 68% of elective cases. Urgent cases received total arterial revascularisation in 57% of cases. However, the emergent cases were suitable for total arterial revascularisation in 17% of cases. Accordingly, combined venous and arterial revascularisations in elective, urgent and emergency operations were 26%, 37% and 50%, respectively (See also Figure 1).

Postoperative course included two patients with a pulmonary embolism and one patient with a peri-operative stroke. Incidentally, the patient who suffered from stroke intra-operatively had an undiagnosed atrial septum defect (ASD), despite pre-operative echocardiography, and received an ASD closure during a later hospital stay after she recovered from the initial operation. 10 patients needed prolonged ventilatory support (>48 h) and six patients developed a transient kidney insufficiency postoperatively (See Table 4).

**Table 4:** **Postoperative course**

Re-Thoracotomy	6 (5)
Bleeding	3 (2)
Respiratory insufficiancy	10 (8)
Postoperative renal insufficiancy	6 (5)
Stroke	1 (1)
Postop coronary angiogram (unplanned)	3 (2)
Secondary thoracic operations	9 (7)
Post-op left ventricular ejection fraction in % (± SD)	56.83 (±12.13)
In hospital death	1 (1)
Revision of bypass grafts	3 (2)

Continuous variables are presented with the standard deviation; categoric variables are presented as number (%).

Three patients needed a revision of their bypass grafts after suffering from postoperative myocardial infarction, and three patients needed revision due to symptomatic bleeding with either a haematothorax (n = 2) or pericardial tamponade (n = 1). One of the patients with postoperative bleeding had undergone a rescue attempt using Abciximab (ReoPro) in the cath lab due to an iatrogenic dissection of the LAD directly prior to operation. Seven patients were re-administered after recovery for removal of the sternal cerclages, all due to pain during movement.

## Discussion

Despite all advances in prevention and diagnostics, as well as general awareness for cardiovascular disease in the elderly, our patient collective with young patients below 50 years of age often present in urgent or emergency settings. We have to assume that especially in these young patients with multiple risk factors; diagnosis of a coronary artery disease is delayed because of inadequate awareness. Clinical presentation of our patients collective was rather heterogeonus which makes a direct, propensity matched comparison very difficult. Especially the high frequency of extra hospital reanimated patients and patients with iatrogenic complications during PCI, makes a comparison to previous published studies for CABG difficult.

Although the recent long-term results from the SYNTAX [1], ASCERT [9], and FREEDOM [10] trials showed significantly better survival rates after CABG than after PCI, CABG rates are declining over the past years, while PCI rates increase accordingly [11]. Nonetheless, CABG remains the Gold Standard for patients with coronary artery disease including those with diabetes and/or complex left main or three-vessel disease [1,9].

The technique of CABG has not changed significantly over the past years. However, the use of bypass material remains under intense discussion. The use of one internal thoracic artery as graft, most often the LITA anastomosed to the LAD combined with venous conduits represents the standard therapy for patients undergoing CABG [12].

Failure rates of up to 12% of saphenous vein grafts within the first week after operation have been described. Therefore alternative grafts such as bilateral internal thoracic artery or radial artery grafts are more frequently used.

The long-term results from recent trials suggest favorable radial artery graft patency rates over saphenous vein grafts [13,14]. Accordingly, several large observational studies have confirmed excellent graft patency and have reported superior long-term survival rates, [15] also after applying propensity matching [4,16] for patients receiving the radial artery as bypass grafts. However, concerns regarding vessel spasm, graft atherosclerosis, and unfavorable results from a number of studies exist. We do however; believe in the use of the radial artery as our standard graft in patients with no contraindications against this approach.

While these young patients would benefit most from a total arterial revascularization given its superior long term patency rates [4,13-16], this approach is frequently not possible. In our series, 57% of urgent and 17% of emergency cases received a TAR in the subgroup analysis.

In our case series, 12% of patients present with a left ventricular ejection fraction of 35% or lower. This underlines the fact that especially this patient collective is administered to the hospital in a later stage of their disease.

**Figure 2:**
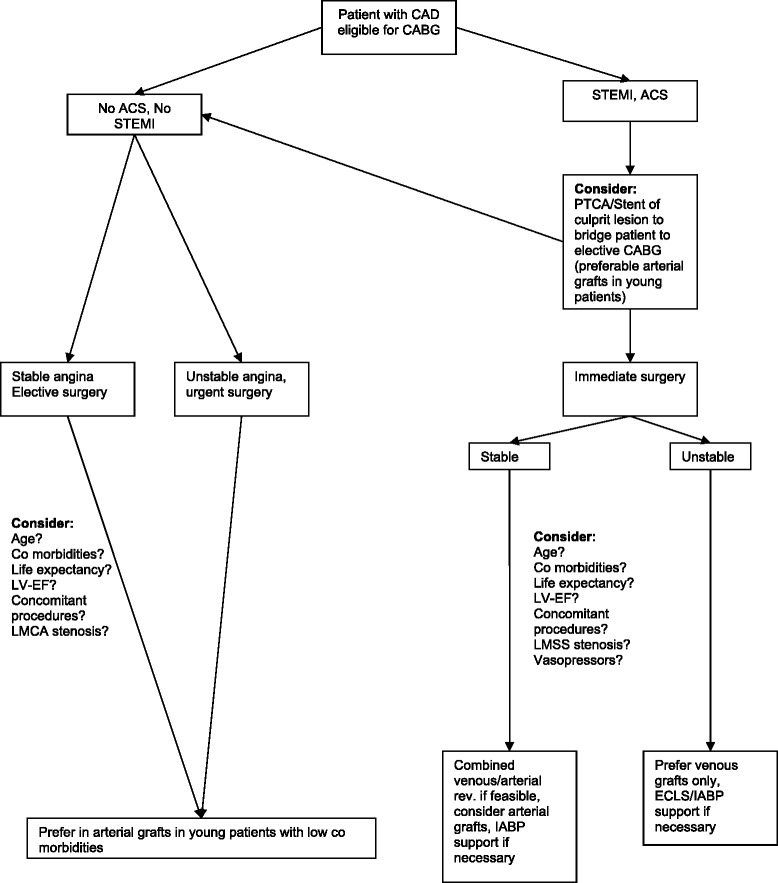
Factual decision making for graft selection in Coronary artery bypass grafting especially in younger patients. ECLS (Extracorporal Life Support System) ACS (Acute Coronary Syndrome), STEMI (ST-elevation myocardial infarction), IABP (intraaortic ballon pump), LV-EF (Lef-ventricular ejection fraction), LMCA (Left main coronary artery).

However, the possible long-term advantage of a TAR is diminished by the fact that the life expectancy of these ill patients is severely diminished. Although, the options for a patient requiring CABG are small (TAR, venous and arterial revascularization, venous revascularization only) the decision making process is rather complex (see Figure 2). In general we agree that absolute contraindications for a TAR are: cardiogenic shock, expected high doses of postoperative catecholamines and a life expectancy of less then 10 years. Graft availability is sometimes limited, too. Especially patients requiring dialysis prior to operation or patients that will require dialysis in the future might, the use of the LITA or RITA is forbidden for sake a cimino fistula. Chronic obstructive lung disease, if severe, might lead to the use of venous grafts only to keep the pleura closed. None the less, we are less concerned about the age of the patient since the use of radial artery grafts for revascularization not only leads to better graft patency rates, however graft harvesting site related complications are also quite rare, and less frequent then with the use of saphenous vein grafts.

Emergency operations, as well as an impaired left ventricular function (below 35% LV-EF) are relative contraindications for a total arterial revascularization. Urgent cases with stable haemodynamics might or might not receive TAR. The usage of TAR in cases of left main coronary artery stenosis (LMCA) is currently under discussion. However, LMCA stenosis is only a relative contraindication for TAR at our department and underlies the surgeon’s discretion.

We also found these patients to be prone for complications, and re-operation rates were relatively high, especially those for removal of sternal cerclages. This could be in fact due to a more active lifestyle of this patient collective. None of these were secondary operations were due to sternal wound infections. In fact, the wound complication rate in this collective was very low. This might be due to the low rate of diabetics, a disease primarily of the elderly patient.

In general our low postoperative mortality rate is similar to that reported by Khawaja et al. [17]. Available data about the postoperative morbidity of young CABG patients report 94% of patients aged <50 years undergoing CABG recovering without any major events and 96% of patients being discharged to home [8].

## Conclusions

Findings prove that surgical revascularization for coronary artery disease can be performed with very low mortality in young patients. However, presentation of patients with high rates of emergency and urgent cases often hinder the favourable approach of total arterial revascularization.

### Limitations

A limitation of this study is the lack of data on long-term outcome. Comparative studies evaluating the immediate and late outcome studies are needed to further refine the strategy of revascularization in young patients receiving CABG.
